# Ethnic‐racial identity in Sweden: Dimensionality, measurement invariance, and psychosocial adjustment

**DOI:** 10.1111/jora.70197

**Published:** 2026-05-17

**Authors:** Tommy Reinholdsson, Ann Frisén, C. Philip Hwang, Linda Juang, Pär D. Stern, Moin Syed

**Affiliations:** ^1^ Department of Psychology University of Gothenburg Gothenburg Sweden; ^2^ Department of Inclusive Education University of Potsdam Potsdam Germany; ^3^ Department of Psychology University of Minnesota Twin Cities Minneapolis USA

**Keywords:** dimensionality, ethnic‐racial identity, generativity, measurement invariance, trust, well‐being

## Abstract

This study examines ethnic‐racial identity (ERI) among majority and minority adolescents in Sweden, focusing on the dimensionality and measurement invariance of ERI subscales, and how the ERI dimensions across these subscales relate to psychosocial adjustment. Using data from 665 Swedish adolescents, we assessed the structure and invariance of six ERI subscales commonly used in ERI research via confirmatory factor analysis. Most subscales showed acceptable structure (with some modifications) and metric invariance across groups, while scalar invariance was fully supported for only one subscale and partially for others. Five ERI dimensions were identified in the subscales: centrality, belonging‐affirmation, clarity, exploration, and public regard. We examined how these dimensions related to well‐being, interpersonal trust, institutional trust, and generativity. Belonging‐affirmation and public regard were positively associated with well‐being in both groups. Among majority youth, belonging‐affirmation was additionally positively associated with interpersonal trust, institutional trust, and generativity, whereas public regard was positively associated with institutional trust and generativity. Among minority youth, public regard was additionally positively associated with interpersonal and institutional trust. Centrality was negatively associated with well‐being and institutional trust among minority adolescents. Exploration was positively associated with generativity in both groups and negatively associated with institutional trust among minority youth. These findings underscore ERI as a psychosocially relevant construct in the Swedish context and highlight how the contextualized experiences of majority and minority adolescents can shape both their interpretation of the ERI construct and its associations with psychosocial adjustment.

## INTRODUCTION

Ethnic‐racial identity (ERI) refers to the aspect of an individual's self‐concept that is derived from their membership in an ethnic‐racial group, and the meaning and significance they attach to that group membership (Ashmore et al., 2004; Rivas‐Drake et al., 2014). Although ERI has been consistently linked to adolescent adjustment in primarily U.S.‐based research, far less is known about how the construct functions in sociocultural contexts where ethnicity and race are less explicitly discussed. Sweden represents such a context. Despite increasing ethnic‐racial diversity, race and ethnicity are often treated as sensitive or avoided topics, reflecting a broader colorblind orientation that leaves adolescents to make sense of ERI in the absence of clear public discourse (Hübinette et al., 2023). Nevertheless, emerging Swedish studies indicate that ERI is salient for youth from a variety of backgrounds (e.g., Abdullahi et al., 2024; Gyberg et al., 2018). At the same time, important questions remain about whether widely used, U.S.‐developed ERI measures capture comparable constructs in Sweden, and how ERI relates to psychosocial adjustment among adolescents in majority and minority positions.

Against this backdrop, the aim of this exploratory study is to expand the growing body of research on ERI by examining the dimensionality and measurement invariance of commonly used ERI subscales and by exploring how the ERI dimensions emerging from these subscales relate to adolescents' well‐being, interpersonal trust, institutional trust, and generativity. By doing so, our purpose is to contribute to an increasingly nuanced understanding of ERI, in general, and specifically in the Swedish context.

### The theoretical foundation of ethnic‐racial identity

The conceptualization of ERI draws upon key elements from three main foundational theories. First, Erikson's psychosocial development theory (1968) emphasizes identity formation as a central task of adolescence, during which individuals strive to establish a coherent and stable sense of self in relation to domains such as ethnicity and race. Second, social identity theory (Tajfel & Turner, 1979) highlights the role of group membership in shaping self‐concept, suggesting that individuals derive part of their identity from their affiliation with ethnic or racial groups, and that in‐group versus out‐group dynamics influence intergroup attitudes and behavior. Third, Stryker's identity theory (Stryker & Burke, 2000) focuses on how identity is shaped through social roles and interactions, suggesting that individuals hold multiple identities that are organized in a hierarchy of salience, being more or less central in a given context.

Consistent with these theoretical perspectives, ERI is not static but develops across the lifespan, shaped by changing contextual, cognitive, and social influences (Williams et al., 2020). Awareness of ethnic‐racial cues emerges in early childhood and becomes more differentiated through the reflective and meaning‐making processes in adolescence. Late adolescence—the focus of this study—represents a transitional period, during which youth remain embedded in school and family contexts while also beginning to navigate the social, academic, and structural changes that characterize emerging adulthood (Umaña‐Taylor et al., 2014).

An important distinction in ERI research, as emphasized in Phinney's (1992) influential work and rooted in social identity theory (Tajfel & Turner, 1979), is the difference between majority and minority group belonging. In the United States, the majority/minority distinction is often framed in terms of being White or non‐White (e.g., Syed & Juang, 2014), whereas in European contexts it is more commonly described in terms of having an immigrant or non‐immigrant background. In the present study, we use the terms *minority* and *majority* youth to distinguish between these two groups. Importantly, the ERI‐related experiences of majority and minority youth are shaped by multiple contextual factors, including social hierarchies, power relations, and the varying salience and complexity of ERI formation. For minority youth in particular, ERI is usually salient and shaped by a need to navigate both heritage and majority cultural identifications (Erentaitė et al., 2018).

Considering the significant impact of context on ERI formation for both minority and majority groups, it is crucial to also consider that much of the work that has established ERI as a key developmental construct has been conducted in the United States. Recent years, however, have seen a growing body of ERI research in Europe (e.g., Erentaitė et al., 2018). In Sweden specifically, a limited amount of research suggests that ERI is developmentally relevant for adolescents (Abdullahi et al., 2024; Svensson & Syed, 2019). However, important questions remain regarding how ERI interacts with the sociocultural context. One such question concerns whether Swedish adolescents interpret and understand ERI differently from their American counterparts, which may have implications for the validity of measures developed in the United States and how ERI dimensions in these measures relate to psychosocial adjustment.

### Sociocultural influences of ERI in the Swedish context

In Sweden, the connotations of ethnicity and race have shifted significantly over the past century. In the early twentieth century, the Swedish State Institute for Racial Biology played a notable role in the development and legitimization of pseudoscientific racism (Ericsson, 2016). Then, after World War II, Sweden instead became a leading international voice for decolonization and anti‐racism, while at the same time a colorblind approach to ethnicity and race became the cultural norm (Hübinette et al., 2023). Although Sweden avoids explicit racial terminology, racialized perceptions nevertheless shape adolescents' experiences, making ERI a relevant construct in this context (Hübinette et al., 2023).

In recent decades, the country has transformed from a predominantly ethnoculturally homogeneous society to one characterized by high diversity: As of 2024, 42% of youth aged 18–29 are either foreign‐born or have at least one parent who was born abroad (Statistics Sweden, n.d.‐a). Recent immigration to Sweden includes both labor migrants from countries such as India and Germany as well as asylum seekers from countries including Ukraine, Syria, Afghanistan, Somalia, and Serbia (Statistics Sweden, n.d.‐b, n.d.‐c). Despite being recognized as a global leader in integration policies, Sweden has experienced growing social tensions related to immigration and cultural pluralism (Petridou et al., 2024; Solano & Huddleston, 2020). These tensions are reflected in the rising influence of the radical right‐wing Sweden Democrats, residential and school segregation, and escalating gang‐related violence in marginalized areas.

In Sweden's diverse cultural landscape, ERI is likely a salient aspect of development for many adolescents, though the challenges they encounter may differ based on their social position. For minority adolescents, ERI development often involves navigating experiences tied to their minoritized status in society, including othering, exclusion, and the need to reconcile multiple cultural influences (Erentaitė et al., 2018). This is reflected in self‐descriptions such as being “another kind of Swede,” highlighting the experience of being positioned outside the majority culture (Gyberg et al., 2018). These dynamics are shaped by a broader European cultural climate, in which diversity is often framed through discourses of “migration” and “foreignness” (Juang et al., 2023). Ethnic majority adolescents in Sweden may also face distinct challenges, such as making sense of what it means to be “Swedish” in a multicultural society (Medin et al., 2025; Svensson & Syed, 2019). They may feel unsure how to relate to cultural diversity and struggle to describe what being Swedish means in a non‐exclusionary way (Lazëri et al., 2024). Thus, while majority adolescents may not encounter the same kinds of identity threat or marginalization as their minority peers, they too engage in a process of ERI formation.

Taken together, Sweden's diverse population, increased immigration in recent decades, and current social tensions associated with immigration may create conditions in which ERI assumes prominence in the identity formation of many young Swedes. Existing research suggests that questions pertaining to ERI make sense to young people in Sweden (e.g., Svensson & Syed, 2019). However, there remains a need to explicitly evaluate the ERI construct in the Swedish context to determine whether Swedish adolescents' perception and interpretation of ERI align with the predominantly U.S.‐based research that underpins how ERI is conceptualized and measured.

### The structural validity of ERI measures

Given the potential influence of context on ERI, it is crucial to evaluate the structural validity of the construct when applied in Sweden, particularly when using scales that were developed in the United States. Two important aspects of such an evaluation involve the assessment of scale dimensionality and measurement invariance across majority and minority group members. Dimensionality, typically evaluated through confirmatory factor analysis (CFA), determines whether a scale adequately captures the intended ERI dimension, as indicated by prior research (Rios & Wells, 2014). CFA is also commonly used to assess measurement invariance, which ensures that the scale functions consistently across subgroups.

### Instruments for measuring ERI

The most common instruments for measuring ERI today include the Multigroup Ethnic Identity Measure (MEIM) (Phinney, 1992), the Ethnic Identity Scale (EIS) (Umaña‐Taylor et al., 2004), and the Multidimensional Inventory of Black Identity (MIBI) (Sellers et al., 1997). These scales measure ERI dimensions that are common across different ethnic‐racial groups, making them suitable for use within and comparisons between ethnic‐racial groups.

#### The multigroup ethnic identity measure (MEIM)

The MEIM (Phinney, 1992; Roberts et al., 1999) conceptualizes ERI as a part of an individual's social identity (Tajfel & Turner, 1979) and is further influenced by Erikson's (1968) psychosocial theory of development and Marcia's (1980) operationalization of Erikson's writings. The MEIM includes two subscales: exploration and commitment. Exploration measures the introspective process of examining one's ethnic background—termed “exploration‐search” for its focus on self‐reflection rather than social engagement (Syed et al., 2013)—while commitment reflects one's sense of belonging and attitudes toward one's ethnic group (Phinney, 1992; Roberts et al., 1999).

Although Phinney initially proposed a single factor, later research supports a two‐factor model distinguishing between exploration and commitment (e.g., Phinney & Ong, 2007). Studies generally support the MEIM's configural and metric invariance across ethnic‐racial groups (e.g., Feitosa et al., 2017), indicating consistency in structure and meaning across groups. However, issues have been reported concerning poor model fit for the exploration‐search subscale in a Swedish study (Abdullahi et al., 2024). Previous research also indicates uncertainty in terms of scalar invariance across majority and minority samples (e.g., Syed & Juang, 2014).

#### The ethnic identity scale (EIS)

Like the MEIM, the Ethnic Identity Scale (EIS; Umaña‐Taylor et al., 2004) draws on social identity theory (Tajfel & Turner, 1979), Erikson's (1968) psychosocial theory, and Marcia's (1980) operationalization of Erikson's model. Building on the MEIM, Umaña‐Taylor and colleagues aimed to clarify the structure of ERI, resulting in three subscales— exploration, affirmation, and resolution—which also form the basis of the EIS short form (EIS‐B; Douglass & Umaña‐Taylor, 2015), which was used in the current study. Exploration measures the extent to which someone has explored their ethnic‐racial background, affirmation measures how they feel about their ERI, and resolution measures the degree to which they have made sense of what their ERI means to them (Umaña‐Taylor et al., 2004). The exploration subscale of the EIS has been described as “exploration‐participation” due to its emphasis on socially oriented exploration (Syed et al., 2013).

The three‐factor structure of the EIS‐B is generally supported (e.g., Douglass & Umaña‐Taylor, 2015), although issues concerning dimensionality have been reported regarding the affirmation subscale of the EIS (Meca et al., 2021). The EIS‐B generally demonstrates configural and metric invariance across diverse ethnic‐racial groups in the United States, but not scalar invariance (e.g., Sladek et al., 2020). In contrast, a Swedish study found support for scalar invariance across minority and majority adolescents for the exploration‐participation and resolution subscales (Abdullahi et al., 2024).

#### The multidimensional inventory of Black identity (MIBI)

The MIBI (Sellers et al., 1997, 1998) conceptualizes ERI differently from the MEIM and EIS, drawing on Sellers et al.'s (1997) Multidimensional Model of Racial Identity (MMRI). This framework builds on aspects of Stryker's identity theory (Stryker & Burke, 2000), Self‐Categorization Theory (Turner & Reynolds, 2011), Nigrescence Theory (Cross, 1994), and collective self‐esteem theory (Crocker et al., 1994). The MIBI operationalizes ERI through three subscales: centrality, private regard, and public regard. Centrality reflects how important ERI is to one's self‐concept; private regard reflects personal feelings about one's group; and public regard reflects one's perceptions of how others view one's group. Originally developed for Black Americans, the MIBI has been adapted and found to be useful in ethnic groups beyond Black Americans (e.g., Abdullahi et al., 2024; Fuligni et al., 2005).

Attempts to confirm the factor structure of the MIBI have mostly failed. In an early test of its construct validity, Cokley and Helm (2001) found poor model fit and concluded that the scale remained in early development, and subsequent studies have raised further concerns about its original structure (e.g., Abdullahi et al., 2024; Vandiver et al., 2009). The MIBI is less widely used across ethnic‐racial groups than the MEIM and EIS‐B. However, there are exceptions: For instance, Yip et al. (2022) found ERI centrality and private regard invariant across African American, Asian American, and Latinx adolescents using a modified factor structure. Similarly, a Swedish study found poor model fit but supported metric (not scalar) invariance across majority and minority youth (Abdullahi et al., 2024).

### ERI and psychosocial adjustment

When exploring ERI in the Swedish context, it is crucial to consider not only factorial structure and invariance but also its association with psychosocial adjustment. Although some European studies have examined the links between ERI and adjustment (e.g., Weber et al., 2021), similar Swedish research is scarce. It is important to address this gap in the literature, because the significance of ERI becomes clearer through its links to outcomes such as well‐being. In the following, we outline the association between ERI and well‐being and discuss its proposed connection to social trust and generativity.

Well‐being is one of the most common outcomes in ERI research, supported by key theoretical frameworks (Erikson, 1968; Stryker & Burke, 2000; Tajfel & Turner, 1979). Among youth in the United States, higher commitment, as measured using the MEIM, is linked to higher well‐being and fewer depressive symptoms (Rivas‐Drake et al., 2014), while resolution also correlates with improved well‐being (Gillespie et al., 2024). Research based in the United States and Austria, respectively, shows that the link between exploration and well‐being is more nuanced (Syed et al., 2013; Weber et al., 2021). Specifically, these studies indicate that exploration‐participation, emphasizing social engagement and measured with the EIS‐B, has been positively linked to well‐being, while exploration‐search, emphasizing a search for meaning tied to ethnicity and race, has been linked to decreased well‐being. The role of centrality in well‐being is similarly nuanced. Some U.S.‐based studies find that centrality strengthens the positive link between affirmation and well‐being among minority adolescents (Brittian et al., 2013) and buffers against the negative effect of discrimination (Cobb et al., 2019). Other research, in the United Kingdom, suggests that centrality might negatively affect well‐being among minority groups in the context of discrimination, but can have a buffering effect among majority groups (Wolfram et al., 2018). Public regard has also been linked to well‐being. Among U.S. youth, higher public regard has been tied to fewer somatic symptoms, while lower public regard may be related to reduced distress over time, a link that may be mediated by preparation for bias (Rivas‐Drake et al., 2009; Willis & Neblett, 2020).

While well‐being, as a broad indicator of psychosocial adjustment, is highly relevant when exploring ERI dimensions in the Swedish context, it is equally important to investigate more specific indicators of adjustment that may be particularly salient in the current sociocultural environment. Erikson's (1968) theory highlights how identity formation in adolescence is shaped by earlier developmental tasks, with basic trust being the foundation of development. Unresolved issues involving trust can thus prevent a coherent identity, which in turn affects later capacities like generativity—referring to one's ability to contribute to others and society (Erikson, 1968). In this study, we examine social trust and generativity as socially embedded indicators of adjustment, potentially relevant in contexts in which polarization, marginalization, and othering may undermine both.

Social trust—defined as the expectation that others will act in fair and predictable ways—is an integral part of both identity development and societal cohesion (Erikson, 1968; Fukuyama, 1995). Typically divided into interpersonal and institutional trust, social trust is shaped by shared norms, incentive structures, and institutional contexts (Fukuyama, 1995). Although research is limited, studies suggest that ERI is associated with trust across groups and contexts. In Sweden, strong group identity based on nationality or neighborhood has been linked to greater trust in institutions corresponding to that social level (e.g., trust in the judicial system at the national level, and in schools at the neighborhood level) (Sohlberg & Esaiasson, 2025). Moreover, for minority adolescents in Norway, feeling accepted and recognized as part of the national community can promote social trust, while experiences of exclusion or bias may erode it (Fretheim et al., 2024). In the United States, ERI centrality among people of color is associated with greater trust in government and media, whereas for White individuals it relates to trust in law enforcement and the courts (Blankenship et al., 2021). A Chinese study found that higher ERI affirmation and belonging, captured in the MEIM, were associated with more positive orientations toward outgroups among both majority and minority youth (Long et al., 2020). Despite these findings, the relationship between ERI and social trust remains underexplored, emphasizing the need for further research to clarify this association.

Generativity, or the drive to contribute to others and society, is another psychosocial capacity linked to identity development (Erikson, 1968). While commonly associated with midlife, it often emerges in adolescence through volunteering, civic engagement, and envisioning one's future roles (Mitchell et al., 2021). Consistent with Erikson's (1968) psychosocial theory, research in the United States shows that a secure sense of identity supports generative concern (Mitchell et al., 2021). U.S. studies further suggest that in contexts where ERI is salient, ERI resolution may fuel generativity (Jensen, 2008; Phan & Kloos, 2023; Santana et al., 2024). Specifically, ERI can inspire civic action among immigrant youth, and ERI socialization predicts civic engagement, mediated by ERI exploration. These findings suggest that ERI could be associated with generative concerns. However, more research is needed to further clarify the link between ERI and this crucial psychosocial capacity.

As outlined above, ERI is linked to psychosocial adaptation at both individual and social levels. Previous research has demonstrated robust associations between various dimensions of ERI and well‐being; however, this relationship remains underexplored in the Swedish context. Moreover, existing research and theoretical frameworks suggest potential links between ERI and broader psychosocial constructs such as social trust and generativity. Although these associations are not yet well studied, exploring them may provide valuable insights into the broader social significance of the ERI construct.

### The current study

While ERI has been established as a significant developmental construct primarily in U.S.‐based research, its applicability and associations in European and especially Swedish contexts remain underexplored. Addressing this is important given Sweden's sociocultural landscape, which is characterized by high ethnic diversity, a colorblind ideology, and social tensions related to cultural pluralism. Against this backdrop, this exploratory study aims to expand the growing body of research on ERI by investigating its dimensional structure, measurement invariance, and relevance for psychosocial adjustment among majority and minority adolescents in Sweden. We address two research questions:

1. Is the dimensionality and measurement invariance of six ERI subscales (centrality, commitment, resolution, exploration‐search, exploration‐participation, and public regard) supported among Swedish ethnic‐racial majority and minority adolescents? This question examines whether the subscales capture their intended ERI dimensions and function equivalently for majority and minority youth.
2. What are the associations between ERI dimensions—captured across these subscales—and indicators of psychosocial adjustment (well‐being, interpersonal trust, institutional trust, and generativity), and do these associations differ between ethnic‐racial majority and minority youth? This question clarifies the subscales' dimensionality and the associations between refined ERI dimensions and psychosocial adjustment.

## METHODS

### Participants and procedures

Data were collected within the research project *Gothenburg Research Study on Ethnic identity and Youth* (GRAY) in spring 2023. Invitations to participate in the study were distributed to principals at thirteen schools in western Sweden. Nine schools agreed to participate. Data were collected by a team of researchers during school hours. As all participants were over the age of 15, parental or guardian consent was not required under Swedish law. Before filling out the questionnaire, participants gave their informed consent. They were also provided a definition of “ethnic belonging” from the Swedish Equality Ombudsman, which encompasses ethnic, racial, and cultural elements (The Equality Ombudsman, n.d.). Participants received a soda and a brownie as a token of appreciation. Ethical approval was granted by the Swedish Ethical Review Authority (reference number: 2022‐06372‐01).

Data were collected from an initial sample of 674 adolescents. Of these, nine were excluded because of missing data relevant to group classification (described below), resulting in an analytic sample of *N* = 665 late adolescents in their last semester of the Swedish equivalent of high school (*M*_age_ = 18.82, *SD* = 0.46, 49.0% female, 49.0% male, 0.9% other). The analytic sample was divided into a majority group (*n* = 313) and a minority group (*n* = 352). This grouping reflects the expectation, discussed earlier, that adolescents' ERI‐related experiences are shaped by different developmental and sociocultural challenges depending on whether they belong to the societal majority group or a minoritized group (Erentaitė et al., 2018; Tajfel & Turner, 1979).

Group assignment was based primarily on participants' self‐reported ERI, which most adolescents (*n =* 613) provided in an open‐ended question. Adolescents who identified solely as Swedish (*n* = 292; 43.3%) were categorized as majority, whereas those who identified as Swedish and at least one additional ERI category (*n* = 181; 26.9%) or exclusively as a non‐Swedish ERI category (*n* = 140; 20.8%) were categorized as minority. The decision to categorize adolescents who identified as Swedish and at least one additional ERI category as part of the minority group was based on the rationale that these youth often encounter experiences of minoritization (see e.g., Gyberg et al., 2018). For the sixty‐one participants who did not provide a valid ERI self‐identification, categorization was based on their own and their parents' country of birth: twenty‐one were classified as Swedish (Swedish‐born with Swedish‐born parents), and thirty‐one as non‐Swedish (born abroad or with two foreign‐born parents). The remaining nine participants did not provide sufficient information and were therefore not categorized.

These procedures resulted in two analytic groups: a majority group consisting of adolescents with solely Swedish ERI, and a minority group consisting of adolescents whose ERI included at least one non‐Swedish background (and may also include Swedish). We acknowledge that both categories are broad and encompass considerable within‐group diversity. Given that this study represents one of the first large‐scale examinations of ERI among late adolescents in Sweden, we prioritized maintaining sufficient statistical power by using broader group definitions. Additional demographic details are provided in Table S1.

Excluding *n* = 61 with missing or otherwise invalid self‐identification data, the adolescents stated their ERI in terms of an association with a nation or geographical area (e.g., “Swedish,” “Sweden,” “Pakistani,” “Africa”) (85.2%), a (more strictly defined) ethnic group (e.g., “Arab,” “Kurdish,” “Assyrian”) (4.4%), in terms of a racial category (e.g., “White,” “Black” (0.8%)), or using a mix of categories pertaining to a nation, ethnicity, and/or racial category (9.5%). In the minority group, *n* = 84 (23.8%) were first‐generation immigrants and *n* = 224 (63.5%) second‐generation. Ethnic‐racial backgrounds and regions were categorized using the UN Geoscheme (United Nations Statistics Division, n.d.). The most common origins in the minority group were Western Asia (30.7%), Southern Europe (18.7%), Northern Europe (12.4%), East Africa (7.2%), and Eastern Europe (6.6%). The remaining (24.4%) were categorized into other geographical areas.

### Measurements

#### Ethnic‐racial identity measures

The ERI subscales included in this study were the MEIM commitment and exploration‐search subscales, the EIS‐B exploration‐participation and resolution subscales, and the MIBI centrality and public regard subscales. These were selected to capture core ERI dimensions while avoiding conceptual redundancy. For example, positive affect toward one's group—assessed in the EIS affirmation and MIBI private regard subscales—is already reflected in the MEIM (Roberts et al., 1999).

##### Exploration‐search (MEIM)

Measured using the five‐item exploration subscale from the Multigroup Ethnic Identity Measure (Phinney, 1992; Roberts et al., 1999), rated on a four‐point Likert scale (1 = strongly disagree, 4 = strongly agree). Example item: “I have spent time trying to find out more about my ethnic group, such as its history, traditions, and customs.” Cronbach's α _majority_ = .66 and α _minority_ = .70.

##### Commitment (MEIM)

Measured using the seven‐item MEIM commitment subscale (Phinney, 1992; Roberts et al., 1999), rated on a four‐point Likert scale (1 = strongly disagree, 4 = strongly agree). Example item: “I have a clear sense of my ethnic background and what it means to me.” Cronbach's α _majority_ = .86 and α _minority_ = .90.

##### Exploration‐participation (EIS‐B)

Measured using the three‐item exploration subscale from the Ethnic Identity Scale‐Brief (Douglass & Umaña‐Taylor, 2015), rated on a four‐point Likert scale (1 = does not describe me at all, 4 = describes me very well). Example item: “I have attended events that have helped me learn more about my ethnicity.” Cronbach's α _majority_ = .77 and α _minority_ = .78.

##### Resolution (EIS‐B)

Measured using the three‐item EIS‐B resolution subscale (Douglass & Umaña‐Taylor, 2015), rated on a four‐point Likert scale (1 = does not describe me at all, 4 = describes me very well). Example item: “I am clear about what my ethnicity means to me.” Cronbach's α _majority_ = .87 and α _minority_ = .87.

##### Centrality (MIBI)

Measured using the eight‐item centrality subscale in the Multidimensional Inventory of Black Identity (Sellers et al., 1997) with modified items to fit any ethnic group (Fuligni et al., 2005), rated on a seven‐point Likert scale (1 = strongly disagree, 7 = strongly agree). Example item: “In general, my ethnicity is an important part of my self‐image.” Cronbach's α _majority_ = .78 and α _minority_ = .74.

##### Public regard (MIBI)

Measured using the six‐item MIBI public regard subscale (Sellers et al., 1998), rated on a seven‐point Likert scale (1 = strongly disagree, 7 = strongly agree). Example item: “Society views my ethnic group as valuable.” Cronbach's α _majority_ = .77 and α _minority_ = .82.

#### Psychosocial adjustment measures

The measures below were tested for metric invariance across the two analytic groups (majority and minority), which was supported. See Supplementary Materials for further details (Table S2).

##### Well‐being

Measured using the five‐item Satisfaction with Life Scale (Diener et al., 1985), rated on a seven‐point Likert scale (1 = strongly disagree, 7 = strongly agree). Example item: “In most ways my life is close to my ideal.” Cronbach's α _majority_ = .80 and α _minority_ = .82.

##### Interpersonal trust

Measured using three items from the European Social Survey (referenced in OECD, 2017), rated on an eleven‐point Likert scale. Anchors for this scale were question specific. Example item: “Generally speaking, would you say that most people can be trusted, or that you can't be too careful in dealing with people?” (0 = you can't be too careful, 10 = most people can be trusted). Cronbach's α _majority_ = .68 and α _minority_ = .73.

##### Institutional trust

Measured using three items from the Australian General Survey (referenced in OECD, 2017) asking about trust in the healthcare system, police, and the justice system. Items were translated into Swedish and rated on a five‐point Likert scale (1 = strongly disagree, 5 = strongly agree). Cronbach's α _majority_ = .71 and α _minority_ = .74.

##### Generativity

Measured using six items from the Loyola Generativity Scale (An & Cooney, 2006; McAdams & de St Aubin, 1992), rated on a four‐point Likert scale (1 = Never, 4 = Almost always). Example item: “You feel like other people need you.” Cronbach's α _majority_ = .81 and α _minority_ = .81.

### Analytic approach

Analyses combined *confirmatory factor analysis* (CFA), *structural equation modeling* (SEM), and *exploratory structural equation modeling* (ESEM) to address two aims: (1) evaluate the dimensionality and measurement invariance of ERI subscales and (2) examine associations between the underlying ERI dimensions, captured across the ERI subscales, and psychosocial adjustment. Analyses were conducted in R version 4.4.2 using the lavaan package (Rosseel, 2012). Models were estimated using robust maximum likelihood (MLR), with robust standard errors and scaled test statistics. Missing data (~2.9%) were handled using full information maximum likelihood (FIML), under the assumption that data were missing at random (MAR).

#### Dimensionality and measurement invariance of ERI subscales

CFA was used to test the dimensionality of each subscale and assess whether the measurement structure was equivalent for majority and minority youth. Because our aim was to evaluate the dimensionality and invariance of each ERI construct as it was originally intended to be used, we tested each subscale individually rather than modeling entire instruments in a single CFA. Testing full multi‐subscale instruments (e.g., all MIBI items simultaneously) would likely result in intercorrelated models in which subscale‐specific issues can be obscured.

Invariance testing followed standard procedures, sequentially evaluating configural, metric, and scalar models, with partial invariance allowed when theoretically justified (Putnick & Bornstein, 2016). Configural invariance indicates that the basic structure of the measure is the same across groups—for example, that the same items cluster together to form the same factors. Metric invariance means that items contribute to the underlying construct in similar ways across groups, which is necessary for comparing associations between constructs across groups. Scalar invariance indicates that item intercepts are equivalent, allowing for meaningful comparisons of latent means. Partial invariance means that some, but not all, items are invariant.

Model fit was evaluated using CFI (≥.95 good, ≥.90 acceptable), RMSEA (≤.06 good, ≤.08 acceptable), and SRMR (≤.05 good, ≤.08 acceptable) (Hu & Bentler, 1999). Changes in fit were evaluated by ΔCFI, ΔRMSEA, and ΔSRMR, with thresholds of ΔCFI = −.01, ΔRMSEA = .015, and ΔSRMR = .030 for metric invariance, and ΔSRMR = .010 for scalar invariance (Chen, 2007; Cheung & Rensvold, 2002).

#### Associations with psychosocial adjustment

Because several ERI subscales contain conceptually overlapping content— particularly MEIM commitment, EIS‐B resolution, and MIBI centrality—we used ESEM rather than traditional CFA/SEM when examining associations with psychosocial adjustment. CFA imposes zero cross‐loadings, which produced highly correlated factors and unstable estimates in preliminary SEM models, making it difficult to interpret the unique contributions of theoretically related ERI components. See Supplementary Materials for further details on this analysis. ESEM provides a more flexible representation of data by allowing cross‐loadings, resulting in ERI dimensions that more accurately reflect the shared and unique variance across subscales (Marsh et al., 2014). This approach produced “cleaned” latent ERI dimensions that accounted for overlap between subscales, enabling clearer tests of associations with well‐being, interpersonal trust, institutional trust, and generativity. It should be noted, however, that the ESEM factors are not strictly equivalent to the CFA‐based factors tested for equivalence across groups. Nevertheless, their close conceptual and empirical similarity supports the validity of comparing groups on these underlying dimensions as well.

ESEM model estimation followed Chou et al. (2025). An initial EFA with geomin rotation provided starting values, and each factor was identified by anchoring one indicator with a strong primary loading. To assess whether the associations between ERI dimensions and adjustment indicators differed across groups, we used a chi‐squared difference test, comparing the unconstrained model to one where the regression paths were constrained to equality across the groups. We also used score tests (via the lavTestScore function in the lavaan r package) to identify which equality constraints on regression paths should be released.

## RESULTS

Means and standard deviations are presented for all key variables (see Table 1). Analyses to assess dimensionality and tests of measurement invariance across majority and minority adolescents are then presented for each scale (see Table 2 for an overview). The factor loadings in the final models can be found in the Table S3. Finally, we present the associations between the ERI dimensions and indicators of psychosocial adjustment (see Table 3 for an overview).

**TABLE 1:** Means and standard deviations for each ERI subscale and psychosocial adjustment indicators.

Variable	*M* (*SD*)	
	Majority	Minority
Exploration‐search (MEIM)	1.84 (0.55)	2.39 (0.70)
Commitment (MEIM)	2.65 (0.61)	3.11 (0.67)
Exploration‐participation (EIS‐B)	1.71 (0.74)	2.28 (0.88)
Resolution (EIS‐B)	2.61 (0.72)	3.07 (0.74)
Centrality (MIBI)	3.54 (0.95)	4.22 (0.97)
Public Regard (MIBI)	5.47 (0.84)	4.64 (1.08)
Well‐being	4.85 (1.04)	4.58 (1.23)
Interpersonal trust	5.29 (1.61)	4.62 (1.74)
Institutional trust	3.52 (0.80)	3.24 (0.85)
Generativity	2.41 (0.57)	2.62 (0.59)

*Note*: All ERI subscales were scored by averaging the items within each subscale after reverse‐scoring where applicable.

**TABLE 2:** Measurement invariance testing for each ERI subscale.

Measure	Model	*χ* ^2^ scaled (df)	Cfi.Robust	Rmsea.Robust (90% CI)	Srmr	Supported fit/invar.
Exploration‐search (MEIM)	Original	49.313 (10)	.920	.111 [.081, .144]	.044	Borderline
	Configural	49.313 (10)	.920	.111 [.081, .144]	.044	Borderline
	Metric	49.646 (14)	.923	.092 [.065, .121]	.047	Yes
	Scalar	117.799 (18)	.791	.134 [.112, .158]	.093	No
	Partial scalar	51.603 (15)	.921	.090 [.064, .118]	.049	Yes
Commitment (MEIM)	Original	210.837 (28)	.897	.160 [.140, .180]	.056	No
	Configural	49.738 (24)	.986	.063 [.037, .088]	.029	Yes
	Metric	64.264 (30)	.982	.064 [.042, .086]	.049	Yes
	Scalar	93.675 (36)	.971	.075 [.057, .094]	.059	No
	Partial scalar	75.636 (35)	.979	.064 [.044, .084]	.054	Yes
EIS‐B (Exploration‐Participation, and Resolution)	Original	24.278 (16)	.995	.041 [.000, .073]	.023	Yes
	Configural	24.278 (16)	.995	.041 [.000, .073]	.023	Yes
	Metric	36.345 (21)	.990	.049 [.019, .075]	.058	Yes
	Scalar	116.704 (26)	.940	.108 [.088, .128]	.132	No
	Partial scalar	36.774 (23)	.991	.045 [.012, .070]	.058	Yes
Centrality (MIBI)	Original	578.296 (40)	.735	.184 [.169, .200]	.096	No
	Configural	89.094 (30)	.963	.080 [.061, .100]	.045	Yes
	Metric	87.441 (36)	.965	.069 [.051, .088]	.048	Yes
	Scalar	138.709 (42)	.926	.093 [.077, .109]	.074	No
	Partial scalar	102.118 (40)	.958	.074 [.054, .086]	.052	Yes
Public regard (MIBI)	Original	40.098 (18)	.982	.064 [.034, .094]	.033	Yes
	Configural	40.098 (18)	.982	.064 [.034, .094]	.033	Yes
	Metric	54.911 (23)	.973	.070 [.044, .096]	.059	Yes
	Scalar	88.780 (27)	.945	.091 [.070, .112]	.080	No
	Partial scalar	65.268 (26)	.964	.074 [.049, .096]	.068	Yes

*Note*: CFI ≥ 0.95, RMSEA ≤0.06, and SRMR ≤0.05 were considered good fit, and CFI ≥0.90, RMSEA ≤0.08, and SRMR ≤0.08 acceptable fit. A meaningful decrease in model fit was indicated by ΔCFI = −.01, ΔRMSEA = .015, and ΔSRMR = .030 for metric invariance, and by ΔSRMR = .010 for scalar invariance.

**TABLE 3:** Associations between ERI dimensions and psychosocial adjustment as standardized beta weights for the majority and minority groups.

ERI dimension	Psychosocial adjustment	Majority	Minority	Score test (*χ* ^2^)^a^
Centrality	Well‐being	−0.07	−0.23*	1.20
	Int. Trust	−0.16	−0.04	1.98
	Inst. Trust	−0.08	−0.25*	1.16
	Generativity	−0.03	−0.01	0.87
Belonging‐affirmation	Well‐being	0.30**	0.41**	**5.99**
	Int. Trust	0.24*	0.05	**5.73**
	Inst. Trust	0.27*	0.11	**5.22**
	Generativity	0.18*	−0.01	3.26
Clarity	Well‐being	0.08	0.03	2.53
	Int. Trust	0.09	−0.08	**7.60**
	Inst. Trust	−0.10	−0.19	1.78
	Generativity	0.10	0.40**	**6.53**
Exploration^b^	Well‐being	0.02	0.14*	**5.22**
	Int. Trust	0.14	−0.05	2.54
	Inst. Trust	0.08	−0.15*	**5.13**
	Generativity	0.26**	0.29**	0.01
Public regard	Well‐being	0.30**	0.31**	0.13
	Int. Trust	0.16	0.26**	0.60
	Inst. Trust	0.23**	0.40**	2.59
	Generativity	0.15*	0.06	0.70

*Note*: Majority group *n* = 313; minority group *n* = 352. Significance is marked in bold (* indicates *p* < .05; ** indicates *p* < .01).

^a^ *χ* ^2^ ≥ 3.84 indicates significant group differences in beta weights.

^b^ The Exploration factor is a combination of Exploration‐search (MEIM) and Exploration‐participation (EIS‐B).

### Dimensionality and measurement invariance

#### Exploration search (MEIM)

The initial five‐item model (Roberts et al., 1999) showed acceptable fit on CFI and SRMR, but not RMSEA. Lacking theoretical justification for correlating high‐loading items, we retained the original structure. Given the borderline fit, we considered configural invariance and dimensionality to be supported in the Swedish context. The metric model, with constrained factor loadings across groups, showed comparable fit, supporting metric invariance. Partial scalar invariance was established by freeing three intercepts. The non‐invariant items were “I am active in organizations or social groups that include mostly members of my own ethnic group,” “I think a lot about how my life will be affected by my ethnic group membership,” and “I participate in cultural practices of my own group, such as special food, music, or customs.”

#### Commitment (MEIM)

The initial commitment model (Roberts et al., 1999) showed poor fit. Modification indices suggested allowing error covariances between two item pairs—one pair reflecting affirmation and the other clarity—both theoretically justifiable given their content overlap. With these covariances, model fit improved and configural invariance was established. The two pairs of items may indicate subdimensions in the model (affirmation and sense of clarity). A subsequent metric model showed comparable fit, supporting metric invariance. Partial scalar invariance was established by freeing one intercept: “I have a lot of pride in my ethnic group and its accomplishments.”

#### Resolution and exploration‐participation (EIS‐B)

Since each subscale included only three items (Douglass & Umaña‐Taylor, 2015), the two were analyzed together to meet the minimum requirement of four indicators for model fit evaluation (Brown, 2006). The two‐factor model showed excellent model fit, supporting configural invariance and the dimensionality of the scale(s) in the Swedish context. A metric model also showed excellent fit, establishing metric invariance. Partial scalar invariance was achieved by freeing all item intercepts for exploration‐participation.

#### Centrality (MIBI)

The initial model (Sellers et al., 1997) showed poor fit. Modification indices suggested correlating the errors of three reverse‐coded items. Thus, a method factor was added, resulting in improved but still poor fit. Modification indices also indicated that the errors of two sets of items should be allowed to covary, which was theoretically justified since all three items involved aspects of belonging. This resulted in a good model fit, supporting configural invariance and scale dimensionality. These error covariances may indicate a subdimension in the model (sense of belonging). The metric model showed similar fit, establishing metric invariance. Partial scalar invariance was established by freeing three intercepts: “In general, my ethnicity is an important part of my self‐image,” “My ethnicity is not a major factor in my social relationships,” and “I have a strong sense of belonging to people from my ethnic group.”

#### Public regard (MIBI)

The initial model, based on Sellers et al. (1998), showed good fit, supporting configural invariance and the construct's dimensionality. The metric model showed comparable fit, establishing metric invariance. Partial scalar invariance was established by freeing one intercept: “My ethnic group is not respected by the broader society.”

### Associations between ERI dimensions and psychosocial adjustment

To examine the associations between ERI dimensions and adjustment, we specified three series of nested models, each focusing on conceptually similar ERI dimensions. Rather than including all ERI factors in a single model, this approach reduced model complexity while allowing for a more nuanced examination of overlapping ERI dimensions.

The first series modeled three factors within the ESEM framework, based on the subscales centrality (MIBI), commitment (MEIM), and resolution (EIS‐B). These dimensions were included in the same model because the previous analysis indicated conceptual overlap among them. In the second series of the ESEM models, we included exploration‐search (MEIM) and exploration‐participation (EIS‐B). The third, focusing on public regard (MIBI), used SEM as it involved only one ERI dimension. For each series, we first specified a multi‐group model in which ERI dimensions predicted four psychosocial adjustment indicators. Group differences were assessed using chi‐squared difference tests and the Lagrange Multiplier test for specific paths.

#### Centrality, belonging‐affirmation, and clarity

Because prior analyses indicated conceptual overlap among ERI centrality (MIBI), commitment (MEIM), and resolution (EIS‐B), we analyzed their associations with psychosocial adjustment using the ESEM framework. A preliminary CFA/SEM model confirmed this overlap, resulting in questionable fit (rCFI = .890, rRMSEA = .057, SRMR = .070), highly correlated factors, and unstable parameter estimates. See Supplementary Materials for details.

The loadings produced in the EFA were analyzed using parallel analysis, showing that a four‐factor solution was most appropriate. See Supplementary Materials for the EFA loadings (Table S4). However, with the fourth factor barely above the random threshold, we proceeded with three factors since we used three subscales. The underlying ERI dimensions that were outlined in the EFA were: (1) centrality, (2) a combination of belonging and affirmation, and (3) clarity.

The ERI dimension labeled centrality consisted mainly of five items from the centrality (MIBI) subscale, with loadings in the range of .47–.58. These included items that appear to capture the core idea of centrality (e.g., “My ethnicity is an important reflection of who I am”).

The ERI dimension labeled belonging‐affirmation consisted mainly of items from commitment (MEIM) and centrality (MIBI). Most of the variance attributed to this factor drew from five items in the commitment (MEIM) subscale, with loadings in the range of .52–.74. These items captured belonging (e.g., “I feel a strong attachment towards my own ethnic group”), affirmation (e.g., “I feel good about my cultural or ethnic background”), or both (e.g., “I am happy that I am a member of the group I belong to”). Two centrality items also loaded strongly on this factor (.83–.84) and related to belonging (“I have a strong sense of belonging with people from my ethnic group” and “I have a strong attachment to other people from my ethnic group”).

The ERI dimension labeled clarity consisted mainly of the three items from the resolution (EIS‐B) subscale (.70–.92) and two items from the commitment (MEIM) subscale (.65–.71). All these items reflected a sense of being clear about what one's ERI means (e.g., “I am clear about what my ethnicity means to me” [EIS‐B] and “I have a clear sense of my ethnic background and what it means to me” [MEIM]).

The ESEM measurement model provided good fit to the data (rCFI = 0.963, rRMSEA = 0.060, SRMR = 0.034). For both the majority and minority groups, the factor covariance for centrality and belonging‐affirmation was low (.01–.13) while the covariance for centrality and clarity was negatively low–moderate for the majority group (−.22) and low for the minority group (.07). Belonging‐affirmation and clarity showed a moderate association in the majority group (.31) and a strong association in the minority group (.72). The ESEM structural model also provided good fit to the data (rCFI = 0.944, rRMSEA = 0.042, SRMR = 0.047).

For the majority group, none of the paths between centrality and the adjustment factors were significant, while all paths were for belonging‐affirmation (well‐being [β = .32, *p* = .001], interpersonal trust [β = .24, *p* = .023], institutional trust [β = .29, *p* = .017], and generativity [β = .19, *p* = .032]). None of the paths between clarity and adjustment were significant for the majority group.

For the minority group, there were significant associations between centrality and well‐being (β = −.28, *p* = .004) and institutional trust (β = −.31, *p* = .006). There was also a significant association for the minority group between belonging‐affirmation and well‐being (β = 0.41, *p* < .001) and between clarity and generativity (β = .39, *p* = .001).

A significant chi‐squared test of difference (*p* = .009) indicated an overall difference between the two groups in terms of these associations and additional score tests indicated significant differences between groups for multiple paths.

#### Exploration‐search and exploration‐participation

Parallel analysis of EFA loadings indicated poor differentiation between the two exploration subscales (MEIM and EIS‐B), confirmed by inflated loadings (>1) and very high factor covariance in the ESEM model. We therefore treated exploration as a single latent factor. Measurement invariance testing supported configural invariance—after allowing error covariance between two EIS‐B items—and metric invariance was also established.

The fit of the SEM measurement model was good (rCFI = 0.950, rRMSEA = 0.077, SRMR = 0.042) and similarly for the structural model (rCFI = 0.910, rRMSEA = 0.053, SRMR = 0.062). For the majority group, the path between exploration and generativity was significant (β = .26, *p* = .003). For the minority group, there were significant paths between exploration and well‐being (β = .14, *p* = .035), institutional trust (β = −.15, *p* = .047), and generativity (β = .29, *p* < .001).

A chi‐squared difference test (*p* = .054) suggested no overall group difference in the effects of exploration on psychosocial adjustment. However, the score tests indicated significant group differences in the paths to well‐being and institutional trust.

#### Public regard

The SEM measurement model showed good fit (rCFI = 0.982, rRMSEA = 0.064, SRMR = 0.033), as did the structural model (rCFI = 0.957, rRMSEA = 0.039, SRMR = 0.049). In the majority group, public regard was significantly associated with well‐being (β = .30, *p* < .001), institutional trust (β= .23, *p* = .005), and generativity (β = .15, *p* = .045). In the minority group, it was significantly associated with well‐being (β = .31, *p* < .001), interpersonal trust (β = .26, *p* = .001), and institutional trust (β = .40, *p* = .001).

A chi‐squared difference test (*p* = .395) indicated no overall group differences in the effects of public regard on psychosocial adjustment, and the score tests confirmed that there were no significant differences in specific paths.

## DISCUSSION

The aim of this study is to expand the growing body of research on ERI by exploring the dimensional structure of commonly used subscales, their measurement invariance, and associations between ERI dimensions, across the subscales, and psychosocial adjustment among majority and minority adolescents in Sweden. The CFA results overall support the dimensionality of the subscales, although acceptable fit for the centrality (MIBI), commitment (MEIM), and exploration (MEIM) scales required minor model modifications or more lenient fit criteria. Metric invariance was established across majority and minority adolescents, with partial support for scalar invariance. The ESEM framework further elucidated the underlying dimensions of ERI, labeled centrality, belonging‐affirmation, clarity, exploration, and public regard. Numerous associations were found between these ERI dimensions and well‐being, interpersonal trust, institutional trust, and generativity, revealing both differences and similarities between groups.

From a broader methodological perspective, these findings provide guidance for future ERI adaptation research, particularly when the goal is to examine how closely related ERI dimensions relate to psychosocial adjustment. In the present study, CFA was appropriate for evaluating whether the original subscales functioned as intended. However, when theoretically adjacent ERI subscales were modeled together to estimate their links with adjustment, CFA/SEM produced highly correlated factors and unstable estimates that obscured their unique contributions. Under such conditions, ESEM can be useful because it allows cross‐loadings, thus reducing bias in factor correlations and structural estimates (Marsh et al., 2014). This property of ESEM can help distinguish content that is relatively specific to a given ERI dimension from content shared across overlapping dimensions, supporting a more refined understanding of ERI. Our findings do not, however, suggest that ESEM should replace CFA/SEM as the default approach in future research. When the original subscales are clearly differentiated, CFA/SEM is preferable because it retains the intended measurement model and facilitates comparison across studies.

### The dimensionality of the ERI subscales in Sweden

The unidimensional structures of the resolution (EIS‐B), exploration‐participation (EIS‐B), and public regard (MIBI) subscales aligned with the data, showing consistency with the theory underlying their operationalization. However, analyses of the exploration‐search (MEIM), centrality (MIBI), and commitment (MEIM) subscales indicate more nuanced or multidimensional constructs.

The exploration‐search (MEIM) subscale demonstrated borderline acceptable model fit, particularly in relation to RMSEA. Given the adequate degrees of freedom and sample size, inflation of RMSEA is unlikely (Kenny et al., 2015). Another possible explanation is that the subscale taps into multiple processes, such as introspective search and social participation (Syed et al., 2013), which might blur the latent construct and reduce fit. However, while this may contribute to the poor fit, modification indices did not clearly indicate that modeling such an overlap would improve fit. A third explanation is that some items may be less relevant or harder to interpret in the Swedish context. Compared with the United States, ERI in Sweden is more implicit, compartmentalized, or even taboo (Hübinette et al., 2023; Svensson & Syed, 2019), which may lead to inconsistent item interpretation and measurement error, for instance, of items that imply an active search for ethnic‐racial meaning. This warrants further investigation, for instance by closely examining how Swedish youth interpret and engage with each exploration‐search item.

Consistent with prior research (e.g., Abdullahi et al., 2024; Cokley & Helm, 2001), the centrality (MIBI) subscale showed poor fit, with belonging emerging as a possible subdimension and negatively worded items clustering together. EFA alongside commitment (MEIM) and resolution (EIS‐B) supported this instability, reinforcing calls for revising the measure. Given that the core construct—how important one's ERI is to one's self‐concept —is conceptually straightforward, a more psychometrically sound version should be attainable. More specifically, the current findings suggest that items tapping into belonging may be conceptually distinct and should be avoided in future revisions. Additionally, the negatively worded items contributed to the subscale's poor fit. While this may reflect method effects, it is also possible that low centrality represents a distinct construct, rather than being the inverse of high centrality. This would be similar to research that distinguishes between, for example, positive and negative body image (Tylka & Wood‐Barcalow, 2015) or positive and negative ERI affirmation (Meca et al., 2021). A low score on centrality (MIBI) may reflect a construct akin to colorblindness—that is, distancing from the idea that ethnicity and race matter (Apfelbaum et al., 2012) —rather than the scale's intended meaning. Considering these and previous results, there is a need for developing improved instruments to capture ERI centrality through further conceptual investigation.

The commitment (MEIM) subscale generally aligned with the data, though some model modifications indicated conceptual nuances. CFA and EFA analyses suggested the presence of three subdimensions: belonging, affirmation, and clarity. While these findings are broadly consistent with the theoretical reasoning that has shaped the operationalization of ERI commitment (e.g., Roberts et al., 1999), whether this structure of ERI commitment aligns with commitment as originally conceptualized by Marcia (1980) remains open to discussion. Marcia (1980) defined commitment as the degree of personal investment in identity choices. Notably, the MEIM item which arguably resembles this conceptualization the most (“I feel a strong attachment toward my own ethnic group”) showed the strongest loading on the commitment factor in the EFA and was the anchor item in the ESEM. Still, the MEIM may underrepresent this personal investment dimension, and if the aim is to capture a more precise commitment construct more explicit items should potentially be considered (e.g., “Being part of my ethnic or racial group shapes my choices and actions”). It could also be argued that treating the subdimensions of ERI commitment as distinct—as belonging, affirmation, and clarity—rather than as a blurred amalgamation is both more useful for research and better reflects the complex nature of ERI.

### Measurement invariance across minority and majority adolescents

Based on the factor structure established in the CFAs, metric invariance was supported across majority and minority adolescents for all subscales, while scalar invariance was generally not supported without modifications (except for ERI resolution). Overall, this aligns with prior research (e.g., Abdullahi et al., 2024; Feitosa et al., 2017; Sladek et al., 2020; Syed & Juang, 2014; Vandiver et al., 2009). This suggests that the relationship between each item and the underlying latent construct is equivalent across Swedish majority and minority adolescents, which enables comparisons of correlations between these ERI subscales and outcomes. However, since scalar invariance was not fully supported (except for EIS‐B Resolution), mean differences between majority and minority adolescents could be biased by differential interpretations of items (Putnick & Bornstein, 2016). This reaffirms the important point raised by Syed and Juang (2014), who noted that the meaning and content of ERI likely differ in important ways between majority and minority groups. These differences may reflect both the broader taboo around discussing ethnicity and race in the Swedish context (Hübinette et al., 2023) and distinct developmental challenges faced by minority and majority adolescents (Erentaitė et al., 2018; Lazëri et al., 2024). This warrants further investigation in future research. It is also further clarified by the associations with psychosocial adjustment as discussed below.

### Associations between ERI dimensions and psychosocial adjustment

#### Centrality, belonging‐affirmation, and clarity

Three dimensions of ERI were outlined in the ESEM, which we labeled centrality, belonging‐affirmation, and clarity.

The belonging‐affirmation dimension emerged as a consistently adaptive aspect of ERI, particularly among members of the majority group. For this group, belonging‐affirmation was positively associated with all four indicators of adjustment (well‐being, interpersonal trust, institutional trust, and generativity). In contrast, among the minority group, a significant association was only found between belonging‐affirmation and well‐being. This notable divergence between the two groups could be explained by the contextualized experiences of majority and minority adolescents in relation to ERI. Specifically, the association between belonging‐affirmation and the societally oriented outcomes (interpersonal trust, institutional trust, and generativity) could reflect the dominant position of the majority group's ERI in Sweden. To clarify, for majority adolescents, feeling positively connected to their ethnic‐racial group may overlap with feeling integrated into society—since their group is highly represented in society—thus the association with social trust and generativity. For minority adolescents, ERI belonging‐affirmation may not equate with societal integration in the same way, thus weakening the association with social trust and generativity. Notably, the link between belonging‐affirmation and well‐being holds for both majority and minority adolescents, suggesting a more universal psychological benefit of feeling affirmed and connected to one's ethnic‐racial group—regardless of societal context.

Centrality emerged in our analysis as a negative ERI dimension, with significant negative associations among minority adolescents in relation to well‐being and institutional trust. This suggests that, for minority youth, a central ERI may be linked to increased psychological strain rather than being a protective factor, in contrast to U.S.‐based research (Cobb et al., 2019). One possible explanation is the challenge of navigating multiple cultural influences in a society where open discussions of ethnicity and race are often avoided (Hübinette et al., 2023). In such colorblind contexts, heightened vulnerability associated with ERI centrality may also stem from lower perceived public regard relative to majority youth.

Broadly, these findings align with U.K. findings linking high centrality to more negative outcomes under conditions of discrimination (Wolfram et al., 2018).

At the same time, several associations with centrality were not significant. For minority adolescents, centrality was not significantly related to interpersonal trust or generativity. One tentative explanation could be that interpersonal trust and generativity may depend more on day‐to‐day relational context than on how central ERI is to one's identity. In contrast, institutional trust is explicitly tied to perceptions of societal structures, where minority adolescents may be more likely to encounter exclusion or bias.

For the majority group, centrality was not significantly linked to any of the adjustment indicators. Unlike minority youth, majority adolescents typically do not experience societal marginalization tied to their ERI, which could explain the lack of association between centrality and their psychosocial adjustment. At the same time, the negative covariance between centrality and clarity may reflect notable developmental challenges for this group. When ERI is more central for majority adolescents, it appears to coincide with lower clarity about what their ERI means. This pattern may align with Phinney's (1989) foreclosure stage of ethnic identity development, in which individuals feel clear about their ERI without exploration or critical reflection. By contrast, higher centrality and lower clarity could reflect how some majority youth struggle to articulate a coherent, inclusive sense of “being Swedish” (Lazëri et al., 2024).

Alongside affirmation—belonging and centrality, ERI clarity exhibited only one significant association with adjustment: a positive association with generativity among minority adolescents—an association that aligns with Erikson's (1968) view of identity clarity as a foundation for generativity. At the same time, clarity was not significantly associated with well‐being, interpersonal trust, or institutional trust in either group. One possibility is that clarity reflects an internal, self‐reflective aspect of identity that primarily shapes future‐oriented motivations when ERI is made salient by context, rather than immediate emotional well‐being or evaluations of others and institutions. In other words, knowing what one's ERI means may provide a sense of direction and agency without necessarily altering one's perceptions of social relationships or societal structures. For the majority group, the lack of significant associations could reflect weaker salience of ERI overall; clarity about one's ERI may simply hold less developmental weight when one's ethnic background is normative and rarely contested. Together, these findings suggest that ERI clarity may function as a contextually dependent, internally oriented resource that promotes future‐oriented engagement.

#### Exploration (search and participation)

Although the constructs were theorized as two separate factors—exploration‐search and exploration‐participation (Syed et al., 2013)—they were not distinguishable in this context. The reason for this could be that we used the short form of the EIS (Douglass & Umaña‐Taylor, 2015), which may have blurred the distinction found using the original EIS (Syed et al., 2013).

The ERI exploration dimension (including both searching and participatory aspects) was associated with generativity across both groups, suggesting that seeking information, reflecting, and engaging with one's ethnic background are associated with broader prosocial or future‐oriented goals. Future research should examine what drives this association. One possibility is that older adolescents experience increasing autonomy and begin to act on it, which may enhance their sense of capability and readiness to contribute to society. For minority adolescents, exploration was also associated with lower institutional trust. This may reflect how exploration exposes minority adolescents to experiences or realizations that challenge their trust in broader societal systems, potentially prompting concerns about generativity and civic engagement. However, there was also a positive link between exploration and well‐being for the minority group, suggesting that while exploration may involve a painful struggle, it is also meaningful and connected to life satisfaction, aligning with the theoretical foundation of identity exploration (Marcia, 1980).

Several associations with exploration were not significant. For minority adolescents, exploration was not significantly related to interpersonal trust. This may reflect that interpersonal trust is shaped more by day‐to‐day social interactions than by identity processes. In this sense, exploring one's ERI may deepen reflections on societal systems (hence the link with institutional trust) without necessarily altering expectations about close others. For majority adolescents, exploration was only associated with generativity and not with trust or well‐being. One possibility is that exploration holds less emotional or social weight for majority youth, for whom ERI is, on average, less externally contested and less tied to experiences of marginalization. As a result, exploration may function primarily as a developmental process tied to purpose and long‐term thinking (as reflected in generativity), without strongly shaping evaluations of social relationships or institutions.

#### Public regard

Public regard—the perception of others' views of one's ethnic‐racial group—was positively linked to well‐being and institutional trust, for both groups of adolescents. For the minority group, it was also linked to higher interpersonal trust, while for the majority group, it was linked to generativity. These patterns present public regard as a potentially adaptive ERI dimension, which corresponds with previous research (Rivas‐Drake et al., 2009). However, it remains to be investigated how these relationships develop over time, with previous research suggesting that lower public regard may buffer against distress over time, mediated by preparation for bias (Willis & Neblett, 2020).

## LIMITATIONS

While this study provides valuable insights into ERI, some limitations should be acknowledged. First, the exploratory, breadth‐over‐depth design prioritized a broad overview of ERI dimensionality and its links to psychosocial outcomes over more detailed analyses, such as potential mediation or interaction effects. Second, the dataset lacked certain relevant ERI subscales, such as affirmation in EIS‐B (Douglass & Umaña‐Taylor, 2015), potentially leaving some aspects of the construct unaddressed. Another limitation concerns the relationship between the CFA and ESEM models. Measurement invariance was tested in CFA, which assumes no cross‐loadings, whereas the ESEM model allows them. As a result, this limits the strict comparability across groups in the ESEM. Nevertheless, the close conceptual and empirical similarity between the ERI subscales and the ERI dimensions supports the validity of comparing the associations between groups. A final limitation concerns the binary categorization of participants into majority and minority groups. While this distinction reflects a meaningful divide, it overlooks the heterogeneity within both groups. For example, the minority group includes youth from diverse ethnic‐racial backgrounds, some of whom may identify more strongly as Swedish than with the other aspect of their ERI, while the majority group may include adolescents with one or more foreign‐born parents. Moreover, some adolescents in the minority group may pass as more Swedish than others due to their appearance, which makes their experience different. Despite these limitations, we believe that the two analytic groups capture a meaningful distinction in how adolescents are positioned within the Swedish ethnic‐racial landscape and therefore remain appropriate for the purposes of the present study. Nevertheless, future research would benefit from a more nuanced analysis that captures within‐group variation in ERI and its associations.

## CONCLUSION

This study affirms ERI as a meaningful psychological construct among adolescents in Sweden, aligning with findings from research conducted in the United States and other cultural contexts. The results overall support the dimensionality of several ERI subscales while also revealing important nuances within the subscales. For instance, ERI commitment (MEIM) appears to comprise three subdimensions: belonging, affirmation, and clarity. These components were tied to overlaps with other ERI subscales—specifically, centrality (MIBI) and commitment (MEIM) shared a belonging dimension, and commitment (MEIM) and resolution (EIS‐B) shared a clarity dimension.

The dimensionality of ERI centrality (MIBI) was further questioned in this study, and we discuss whether low centrality, to some extent, may be conflated with the concept of colorblindness. Since the scale's dimensionality has been questioned before, in both Swedish‐ and U.S.‐based research (Abdullahi et al., 2024; Vandiver et al., 2009), we recommend using an alternative measure of ERI centrality along with further conceptual exploration.

The inclusion of both an intrapsychic outcome (well‐being) and societally oriented outcomes (interpersonal trust, institutional trust, and generativity) highlights the interaction of ERI dimensions and the contextualized experiences of majority and minority adolescents. The negative association between the centrality dimension and well‐being and institutional trust among minority adolescents may reflect their experience of marginalization in Swedish society, as may the negative link between exploration and institutional trust. In this group, ERI clarity may function as an internally oriented resource that promotes future‐oriented engagement, as reflected in generativity. Conversely, in the majority group, the positive links between the ERI dimension of belonging‐affirmation and interpersonal trust, institutional trust, and generativity may reflect that their ERI is broadly aligned with and affirmed by the prevailing social and cultural institutions of Swedish society. At the same time, the negative relationship between centrality and clarity among the majority adolescents suggests that some majority youth may struggle to make sense of their ERI.

Consistent with prior research, ERI was positively associated with well‐being for both groups—particularly through the dimensions of belonging‐affirmation and public regard, both of which reflect a sense of esteem—personal in the case of belonging‐affirmation and collective in the case of public regard. Another point of convergence was in the positive association between ERI exploration and generativity. These findings reinforce the role of ERI as a component of adolescent well‐being, regardless of adolescents' minority or majority group membership. Furthermore, the link between exploration and generativity suggests that engaging with one's ethnic‐racial background may be tied to a future‐oriented mindset and a desire to contribute to others, highlighting the broader significance of ERI.

Overall, the divergences and commonalities in these findings underscore the relevance of ERI as a psychological construct in Sweden, while adding nuance by revealing and unpacking complexities within its dimensions. The results further indicate that associations with adjustment are shaped by whether adolescents belong to a minority or majority group. These insights call for more inclusive social structures and affirm that exploring one's ethnic‐racial background holds value for youth of both minority and majority backgrounds in diverse societies.

## AUTHOR CONTRIBUTIONS

**Ann Frisén:** Conceptualization; funding acquisition; writing – review and editing; project administration; supervision; methodology. **Tommy Reinholdsson:** Conceptualization; investigation; writing – original draft; methodology; formal analysis; project administration; data curation; software. **Linda Juang:** Writing – review and editing. **Pär D. Stern:** Investigation; writing – review and editing; project administration; methodology. **Moin Syed:** Methodology; writing – review and editing; supervision; conceptualization. **C. Philip Hwang:** Writing – review and editing; methodology.

## FUNDING INFORMATION

This study was funded by the Sten A Olsson Foundation for Research and Culture. The funding body had no role in the study design; data collection, analysis, or interpretation; or the writing of the manuscript.

## CONFLICT OF INTEREST STATEMENT

The authors have no conflicts of interest to disclose.

## ETHICS STATEMENT

Ethical approval was granted by the Swedish Ethical Review Authority on December 14, 2022 (reference number: 2022–06372‐01).

## CONSENT

Informed consent was obtained from all participants. As all participants were over the age of 15, parental or guardian consent was not required under Swedish law.

## Supporting information


Table S1.

## Data Availability

The data that support the findings of this study are available on request from the corresponding author. The data are not publicly available due to privacy or ethical restrictions.
