# Supplementary material for: Ethnic‐racial identity in Sweden: Dimensionality, measurement invariance, and psychosocial adjustment
Source: J Res Adolesc. 2026 May 17;36:e70197. doi: 10.1111/jora.70197 (PMC13181231; doi:10.1111/jora.70197)
Supplement: Supplementary file 1 — Table S1. [file JORA-36-0-s001.docx]

**Supplementary Materials**

Table of Content

[Table S1. Demographic characteristics of the analytic sample and analytic groups at T1 2](#_Toc226554937)

[Table S2: Measurement invariance testing for the collective set of psychosocial adjustment indicators 3](#_Toc226554938)

[Table S3: Factor loadings for all items in all ERI subscales for the majority group and the minority group 4](#_Toc226554939)

[Table S4. EFA factor loadings for the items in Centrality (MIBI), Commitment (MEIM) and Resolution (EIS-B) 6](#_Toc226554940)

[Centrality, Belonging–Affirmation, and Resolution (Supplementary SEM) 8](#_Toc226554941)

[Preliminary SEM (confirming overlap) 8](#_Toc226554942)

# Table S1. Demographic characteristics of the analytic sample and analytic groups at T1

| **Table S1.** |  |  |  |
| --- | --- | --- | --- |
| Demographic characteristics of the analytic sample and analytic groups at T1 | | |  |
|  | All (*N* = 665) | Majority (*n* = 313) | Minority (*n* = 352) |
| Age in years, M (SD) | 18.82 (0.46) | 18.80 (0.38) | 18.83 (0.54) |
| Gender ^a^ |  |  |  |
| Male (%) | 49.9 | 51.8 | 48.3 |
| Female (%) | 49.0 | 47.3 | 50.6 |
| Other (%) | 0.9 | 1.0 | 0.9 |
| Born in Sweden (%) | 86.9 | 98.7 | 76.4 |
| Generational Status ^b^ |  |  |  |
| First Generation (%) | 13.4 | 1.6 | 23.8 |
| Second Generation (%) | 35.7 | 4.5 | 63.5 |
| Citizenship |  |  |  |
| Swedish only (%) | 80.8 | 95.8 | 68.5 |
| Swedish and other (%) | 13.2 | 1.9 | 23.5 |
| Other only (%) | 1.8 | 0.0 | 3.4 |
| Self-Assessed SES, M (SD) ^c^ | 3.26 (0.75) | 3.39 (0.66) | 3.15 (0.81) |
| *Note.* |  |  |  |
| ^a^ Percentages for gender may not sum to 100 due to rounding and one missing gender response in the minority group. | | | |
| ^b^ First- and second-generation status were derived from participants’ and parents’ country of birth, whereas majority/minority grouping was based primarily on adolescents’ self-reported ethnic-racial identity. These classifications therefore do not fully overlap. Because information on grandparents’ country of birth was not collected, third-generation-or-higher status could not be reliably identified and was therefore not reported as a separate category. | | | |
| ^c^ Socioeconomic status (SES) was operationalized using a single-item measure of subjective financial situation (Williams et al., 2017), with response options ranging from 1 = do not meet basic expenses to 4 = live comfortably. | | | |

# Table S2: Measurement invariance testing for the collective set of psychosocial adjustment indicators

| **Table S2**  *Measurement invariance testing for the collective set of psychosocial adjustment indicators* | | | | | |  |
| --- | --- | --- | --- | --- | --- | --- |
|  |  |  |  |  |  |  |
| Measure | Model | χ2 scaled (df) | cfi.robust | rmsea.robust (90% CI) | srmr |  |
|  |  |  |  |  |  |  |
| Well-being, Interpersonal Trust, Institutional Trust, and Generativity | Configural | 322.283 (196) | 0.96 | .047 [.037, .056] | .047 |  |
|  | Metric | 329.612 (211) | 0.957 | .044 [.034, .053] | .052 |  |
| *Note.* Well-being (5 items: Satisfaction with Life Scale ([Diener, 1985]), Interpersonal Trust (3 items from the European Social Survey [referenced in OECD, 2017]), Institutional Trust (3 items from the Australian General Survey [referenced in OECD, 2017]) and Generativity (6 items from the Loyola Generativity Scale (An & Cooney, 1992; McAdams & de St. Aubin, 1992)). | | | | | |  |
|  |  |  |  |  |  |  |
|  |  |  |  |  |  |  |
|  |  |  |  |  |  |  |

# Table S3: Factor loadings for all items in all ERI subscales for the majority group and the minority group

| **Table S3** |  |  |  |
| --- | --- | --- | --- |
| *Factor loadings for all items in all ERI subscales for the majority group and the minority group.* | | | |
|  |  | Loading | |
| ERI Subscale | Item | Majority | Minority |
| Exploration-search (MEIM) – Partial Scalar Invariance | 1. have spent time trying to find out more about my ethnic group, such as its history, traditions, and customs. | .61 | .74 |
| The intercepts of item 2, 3 and 5 were non-invariant. | 2. I am active in organizations or social groups that include mostly members of my own ethnic group. | .45 | .57 |
|  | 3. I think a lot about how my life will be affected by my ethnic group membership. | .40 | .39 |
|  | 4. In order to learn more about my ethnic background, I have often talked to other people about my ethnic group | .71 | .70 |
|  | 5. I participate in cultural practices of my own group, such as special food, music, or customs. | .39 | .49 |
|  |  |  |  |
| Commitment (MEIM) – Partial Scalar Invariance | 1. I have a clear sense of my ethnic background and what it means for me. | .64 | .57 |
| The intercept of item 5 was non-invariant. | 2. I am happy that I am a member of the group I belong to. | .63 | .58 |
|  | 3. I have a strong sense of belonging to my own ethnic group. | .84 | .80 |
|  | 4. I understand pretty well what my ethnic group membership means to me. | .77 | .66 |
|  | 5. I have a lot of pride in my ethnic group and its accomplishments | .71 | .63 |
|  | 6. I feel a strong attachment towards my own ethnic group. | .86 | .87 |
|  | 7. I feel good about me cultural or ethnic background | .67 | .58 |
|  |  |  |  |
| Exploration-participation & Resolution (EIS-B) – Partial Scalar Invariance | 1. I have attended events that have helped me learn more about my ethnicity. | .72 | .79 |
| The intercepts of the exploration-participation item 1, 2 and 3 were non-invariant. | 2. I have read books/magazines/newspapers or other materials that have taught me about my ethnicity. | .59 | .62 |
|  | 3. I have participated in activities that have taught me about my ethnicity. | .79 | .87 |
|  |  |  |  |
|  | 1. I am clear about what my ethnicity means to me. | .79 | .77 |
|  | 2. I know what my ethnicity means to me. | .88 | .93 |
|  | 3. I have a clear sense of what my ethnicity means to me. | .79 | .83 |
|  |  |  |  |
| Centrality (MIBI) – Partial Scalar Invariant Model | 1. In general, my ethnicity is an important part of my self-image. | .76 | .79 |
|  | 2. I have a strong sense of belonging to people from my ethnic group. | .64 | .57 |
|  | 3. My ethnicity is not a major factor in my social relationships. (R) | .17 | .13 |
|  | 4. I have a strong attachment to other people from my ethnic group. | .60 | .55 |
|  | 5. My ethnicity is not important to my sense of what kind of person I am. (R) | .53 | .44 |
|  | 6. My destiny is tied to the destiny of other people of my ethnicity. | .41 | .34 |
|  | 7. My ethnicity is an important reflection of who I am. | .86 | .85 |
|  | 8. Overall, my ethnicity has very little to do with how I feel about myself. (R) | .21 | .19 |
|  |  |  |  |
| Public Regard (MIBI) – Partial Scalar Invariance | 1. Society views my ethnic group as valuable. | .52 | .43 |
| The Intercept of item 3 was non-invariant. | 2. In general, other groups view my ethnic group in a positive manner. | .85 | .75 |
|  | 3. My ethnic group is not respected by the broader society | .58 | .50 |
|  | 4. Overall, my ethnic group is considered good by others. | .83 | .80 |
|  | 5. In general, others respect my ethnic group. | .85 | .83 |
|  | 6. On average, most people consider my ethnic group to be less effective than other ethnic groups. | .42 | .36 |
| *Note*. All factor loadings were statistically significant *p* < .01. | | | |

# Table S4. EFA factor loadings for the items in Centrality (MIBI), Commitment (MEIM) and Resolution (EIS-B)

| **Table S4** | | | | |
| --- | --- | --- | --- | --- |
| *EFA factor loadings for the items in Centrality (MIBI), Commitment (MEIM) and Reslution (EIS-B).* | | | | |
|  |  | EFA Factors (labelled) | | |
| ERI Subscale |  | Affirmation-belonging | Clarity | Centrality |
| Commitment (MEIM) | 1. I have a clear sense of my ethnic background and what it means for me. | 0.12 | 0.71 | 0.05 |
|  | 2. I am happy that I am a member of the group I belong to. | 0.63 | 0.09 | -0.13 |
|  | 3. I have a strong sense of belonging to my own ethnic group. | 0.71 | 0.13 | -0.01 |
|  | 4. I understand pretty well what my ethnic group membership means to me. | 0.27 | 0.65 | -0.06 |
|  | 5. I have a lot of pride in my ethnic group and its accomplishments | 0.52 | 0.21 | 0.05 |
|  | 6. I feel a strong attachment towards my own ethnic group. | 0.74 | 0.15 | 0.01 |
|  | 7. I feel good about me cultural or ethnic background | 0.57 | 0.19 | -0.19 |
| Centrality (MIBI) | 1. In general, my ethnicity is an important part of my self-image. | 0.53 | 0.03 | 0.47 |
|  | 2. I have a strong sense of belonging to people from my ethnic group. | 0.83 | -0.15 | 0.01 |
|  | 3. My ethnicity is not a major factor in my social relationships. (R) | -0.05 | -0.02 | 0.51 |
|  | 4. I have a strong attachment to other people from my ethnic group. | 0.84 | -0.17 | 0.03 |
|  | 5. My ethnicity is not important to my sense of what kind of person I am. (R) | 0.18 | 0.03 | 0.58 |
|  | 6. My destiny is tied to the destiny of other people of my ethnicity. | 0.41 | -0.13 | 0.13 |
|  | 7. My ethnicity is an important reflection of who I am. | 0.59 | 0.00 | 0.44 |
|  | 8. Overall, my ethnicity has very little to do with how I feel about myself. (R) | -0.06 | 0.01 | 0.54 |
| Resolution (EIS-B) | 1. I am clear about what my ethnicity means to me. | 0.11 | 0.70 | 0.08 |
|  | 2. I know what my ethnicity means to me. | -0.06 | 0.92 | 0.03 |
|  | 3. I have a clear sense of what my ethnicity means to me. | -0.01 | 0.87 | -0.03 |
|  | | | | |

# Centrality, Belonging–Affirmation, and Resolution (Supplementary SEM)

**Because several ERI subscales contain conceptually overlapping content – particularly MEIM commitment, EIS-B resolution, and MIBI centrality – we used ESEM rather than traditional CFA/SEM when examining associations with psychosocial adjustment. CFA imposes zero cross-loadings, which produced highly correlated factors and unstable estimates in preliminary SEM models, making it difficult to interpret the unique contributions of theoretically related ERI components.**

## Preliminary SEM (confirming overlap)

We estimated a multi-group SEM using robust maximum likelihood estimation (MLR) with full information maximum likelihood (FIML) to handle missing data. The model included latent factors for commitment (MEIM), centrality (MIBI), and resolution (EIS-B), as well as latent factors for psychosocial adjustment outcomes (well-being, interpersonal trust, institutional trust, and generativity). Overall model fit was questionable, with a robust CFI of .890, a robust RMSEA of .057 (90% CI [.053, .061]), and an SRMR of .070.

The SEM specification produced very high latent correlations among the ERI factors, consistent with conceptual overlap and raising concerns about multicollinearity and suppression effects. In the minority group, the standardized latent covariances were .828 between commitment and centrality, .893 between commitment and resolution, and .659 between centrality and resolution. In the majority group, the corresponding covariances were .670, .531, and .234. This pattern suggests that when cross-loadings were constrained to zero, shared item content across ERI subscales was absorbed into the factor covariances, inflating associations among the latent constructs and undermining their discriminant validity.

The high degree of overlap among the ERI factors in the SEM framework was also reflected in the structural regression estimates predicting psychosocial adjustment. Most notably the extremely large standardized effect of commitment on well-being in the minority group (β = .93) suggested suppression or instability arising from the strong intercorrelations among ERI factors.

Taken together, these findings indicate that the SEM framework was not appropriate for disentangling the unique contributions of centrality, commitment, and resolution in this dataset.

# References

Williams, V. F., Smith, A. A., Villanti, A. C., Rath, J. M., Hair, E. C., Cantrell, J., ... & Vallone, D. M. (2017). Validity of a subjective financial situation measure to assess socioeconomic status in US young adults. *Journal of Public Health Management and Practice*, *23*(5), 487-495.
